# DNA replication and transcription programs respond to the same chromatin cues

**DOI:** 10.1101/gr.160010.113

**Published:** 2014-07

**Authors:** Yoav Lubelsky, Joseph A. Prinz, Leyna DeNapoli, Yulong Li, Jason A. Belsky, David M. MacAlpine

**Affiliations:** Pharmacology and Cancer Biology, Duke University Medical Center, Durham, North Carolina 27710, USA

## Abstract

DNA replication is a dynamic process that occurs in a temporal order along each of the chromosomes. A consequence of the temporally coordinated activation of replication origins is the establishment of broad domains (>100 kb) that replicate either early or late in S phase. This partitioning of the genome into early and late replication domains is important for maintaining genome stability, gene dosage, and epigenetic inheritance; however, the molecular mechanisms that define and establish these domains are poorly understood. The modENCODE Project provided an opportunity to investigate the chromatin features that define the *Drosophila* replication timing program in multiple cell lines. The majority of early and late replicating domains in the *Drosophila* genome were static across all cell lines; however, a small subset of domains was dynamic and exhibited differences in replication timing between the cell lines. Both origin selection and activation contribute to defining the DNA replication program. Our results suggest that static early and late replicating domains were defined at the level of origin selection (ORC binding) and likely mediated by chromatin accessibility. In contrast, dynamic domains exhibited low ORC densities in both cell types, suggesting that origin activation and not origin selection governs the plasticity of the DNA replication program. Finally, we show that the male-specific early replication of the X chromosome is dependent on the dosage compensation complex (DCC), suggesting that the transcription and replication programs respond to the same chromatin cues. Specifically, MOF-mediated hyperacetylation of H4K16 on the X chromosome promotes both the up-regulation of male-specific transcription and origin activation.

Every cell cycle, the eukaryotic genome is duplicated during S phase by the activation and progression of hundreds to thousands of bidirectional DNA replication forks. Start sites of DNA replication, termed origins, are not all activated simultaneously during entry into S phase, but rather in a coordinated and temporal manner, resulting in specific sequences replicating at discrete times (Schwaiger and Schubeler 2006). The advent of genome-wide technologies has made it possible to survey the replication timing program from multiple eukaryotic organisms (Raghuraman et al. 2001; Schubeler et al. 2002; Jeon et al. 2005; Hiratani et al. 2008). All genomes surveyed to date exhibit a specific and reproducible replication timing program. Recent studies have demonstrated that the ordered duplication of the genome contributes to genome stability (Donley and Thayer 2013), epigenetic inheritance (Lande-Diner et al. 2009), gene dosage (Nordman and Orr-Weaver 2012), and mutational frequency (Stamatoyannopoulos et al. 2009; Agier and Fischer 2012; Weber et al. 2012). However, the molecular mechanisms by which the replication timing program is defined and regulated are poorly understood.

The time at which a sequence replicates is dependent on origin selection and activation. Start sites of DNA replication are marked by the origin recognition complex (ORC) which, together with Cdt1 and Cdc6, coordinates the loading of the replicative helicase Mcm2-7 complex to form the pre-replicative complex (pre-RC) in G1 (Bell and Dutta 2002). The density of ORC-associated sequences along the chromosome is correlated with replication timing, with regions of high ORC density replicating earlier in S phase than regions of low ORC density (MacAlpine et al. 2010). The correlation between ORC density and replication timing is likely due to an increased density of potential replication origins. Finally, the potential of an origin to initiate during S phase is governed by limiting replication factors required for origin activation (Mantiero et al. 2011; Tanaka et al. 2011). Each of these factors—origin selection and activation—are mediated in part by the local chromatin environment and genome organization.

Chromatin can be broadly classified into two distinct states—euchromatin and heterochromatin. The euchromatin is gene rich and marked by the presence of activating post-translational histone modifications including acetylation and methylation of specific lysine residues (Rando 2012). In contrast, the heterochromatin is gene poor and those genes that do reside in heterochromatin are frequently repressed by specific histone modifications including H3K9 and H3K27 methylation. Early replication studies noted that progression through S phase was not uniform across the genome but rather that the euchromatin and heterochromatin were replicated at distinct times during S phase (Stambrook and Flickinger 1970). Early replicating regions were associated with transcriptionally active regions of the euchromatin, whereas late replicating regions were associated with inactive regions and gene-poor heterochromatin. These results suggest that the transcription and replication programs respond to similar chromatin embedded cues.

Most recently, genome-wide studies in a number of eukaryotic organisms have extended the correlation between DNA replication and transcriptional activity beyond just the classification of euchromatin and heterochromatin. One of the first genome-wide replication timing studies in a metazoan identified broad early and late replicating domains (>100 kb) across the euchromatic portion of the *Drosophila* chromosome arms (Schubeler et al. 2002). A significant correlation between gene expression and time of replication was identified, with actively transcribed genes being replicated early in S phase. Although the correlation between gene expression and replication timing is relatively weak at the level of individual genes, it is much stronger when integrated over broad chromosomal domains (∼150 kb). This suggests that it is the integration of the chromatin features associated with active transcription over broad domains that influences the DNA replication program (MacAlpine et al. 2004). Recent work in mammalian systems has identified a strong link between genome architecture, derived from three-dimensional chromatin interaction maps, and replication, suggesting that the higher order organization of the genome in the nucleus is also an important modulator of the transcription and DNA replication programs (Ryba et al. 2010).

The replication program is dynamic, responding to developmental and cell-type-specific signals. Embryonic cells replicate their entire genome in a short time period from a large number of randomly placed origins (Blumenthal et al. 1974). This pattern changes at the mid-blastula transition when S-phase length increases and the replication program is established (Newport and Kirschner 1982; Hyrien et al. 1995; McCleland et al. 2009). The analysis of replication timing in pluripotent stem cells undergoing differentiation has revealed that different cell-type-specific lineages change their gene expression pattern and chromatin architecture as well as their pattern of replication timing (Hiratani et al. 2010; Ryba et al. 2010; Chandra et al. 2012). These developmentally programmed changes in DNA replication timing indicate a plasticity in the ability to define and activate potential replication origins from the same sequence elements (Gilbert 2005). Changes in non-CpG promoter activity and chromatin accessibility have been associated with changes in the replication program during differentiation (Hiratani et al. 2010; Takebayashi et al. 2012), but a link between specific chromatin modifications has yet to be established. In addition, it is still unclear whether these lineage-specific changes in DNA replication are mediated by changes in origin selection (ORC binding), origin activation, or both.

Here we describe the replication program of three *Drosophila* modENCODE cell lines using next-generation sequencing (Hansen et al. 2010). Integration of the replication timing data with other modENCODE data types including histone modifications, DNA-binding proteins, and gene expression (The modENCODE Consortium et al. 2010) not only allows us to identify chromatin features that are associated with early and late replication, but also provides an opportunity to identify changes in the chromatin landscape that are associated with cell-type-specific replication patterns. We find that early and late replicating domains are differentiated by the enrichment of activating and repressive histone modifications, respectively. Analysis across the three cell lines allowed us to identify static domains that were early or late in all cell lines as well as dynamic domains that changed their replication timing. ORC density is highest in static early replicating domains, suggesting that the density of ORC binding and the selection of potential origins is an important determinant of the DNA replication program. In contrast, dynamic domains, those that switch from early to late replicating or vice versa, exhibit chromatin signatures that are similar to those found in the static late replicating domains and exhibit low ORC binding regardless of the time of replication. These results suggest that the replication program for static early and late replicating domains is primarily established by origin selection (ORC binding) and that origin activation modulates the plasticity of the DNA replication program between cell lines. Finally, we show that the X chromosome and male-specific patterns of early replication (Berendes 1966; Lakhotia and Mukherjee 1970; Meer 1976; Schwaiger et al. 2009) are dependent on the dosage compensation complex (DCC) and that a single histone modification is sufficient to alter the replication timing of an entire chromosome.

## Results

### Replication timing profiles of three *Drosophila* cell lines

The modENCODE Consortium has extensively characterized the transcription, replication, and chromatin landscape of three *Drosophila* cell lines. These cell lines are routinely used by the research community for a variety of functional studies and are representative of different tissues isolated from male and female flies. Kc167 (Echalier and Ohanessian 1969) cells were derived from female embryos, S2 (Schneider 1972) cells were derived from male embryos, and DmBG3 (Ui et al. 1994) cells were derived from the central nervous system of male third instar larva. Previously, we used synchronized populations of cells and tiling microarrays to generate replication timing profiles for each of these cell lines (Eaton et al. 2011). In our earlier work, we synchronized the cells in early S phase by treatment with the drug hydroxyurea (HU), and then released the cells into S phase. A potential caveat of these experiments was that treatment with HU, which depletes nucleotide pools and activates the intra-S-phase checkpoint, may impact the normal DNA replication program (Anglana et al. 2003; Karnani and Dutta 2011; Poli et al. 2012). To ensure that we were examining an unperturbed DNA replication program, we have most recently used cell sorting and next-generation sequencing (Repli-Seq) (Hansen et al. 2010) to profile the replication dynamics of three modENCODE *Drosophila* cell lines in an unbiased manner.

**Figure 1.**
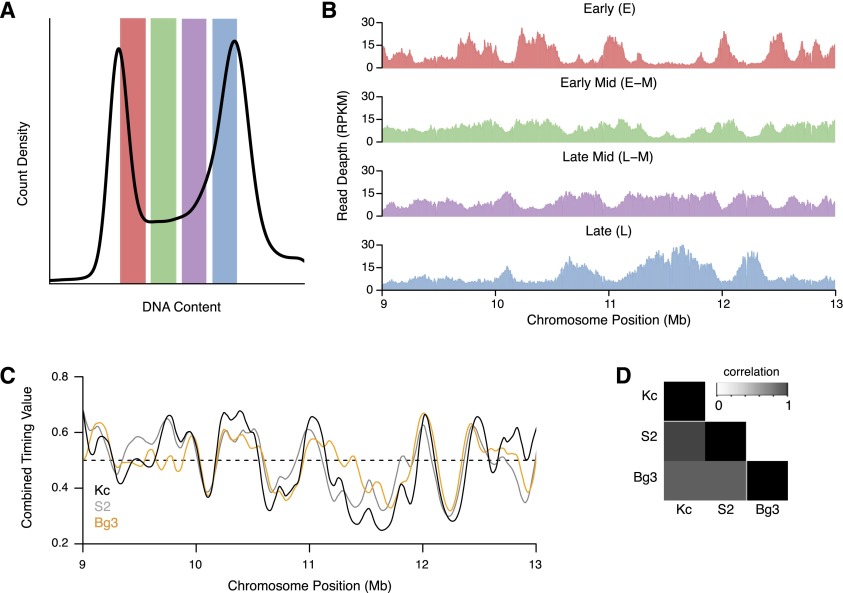
Generation of replication timing profiles. (*A*) Repli-Seq cell sorting. BrdU pulse-labeled cells were sorted into four S-phase fractions by flow cytometry: early, early-mid, mid-late, and late. BrdU-labeled DNA was isolated from the flow-sorted cells and active replication intermediates were precipitated with anti-BrdU antibodies and sequenced by next-generation sequencing. (*B*) Distribution of BrdU-labeled sequences in Kc167 cells. The read depth (RPKM) from each fraction is shown for a 4-Mb portion of chromosome 2L. The early (E, red) and late (L, blue) fractions are enriched in largely nonoverlapping locations with the middle fractions (early-mid [E–M], green; late-mid [L–M], purple) displaying an intermediate pattern changing from E to L. (*C*) Replication timing profiles for Kc167 (black), S2 (gray), and DmBg3 (orange) cells. Replication timing ratios were derived from the four fractions to generate a relative timing value for each genomic position. High values represent early replicating regions and low values represent late replicating regions. A representative 4-Mb region on chromosome 2L is shown. (*D*) Replication profiles are conserved but not identical between cell lines. Pairwise Pearson correlation for the replication timing values was determined for each pair of cell lines.

Actively replicating cells from an asynchronous population were pulse-labeled with the nucleotide analog BrdU for 1 h. The cells were then sorted by flow cytometry into four populations, representing early, mid-early, mid-late, and late S phase, based on their DNA content (Fig. 1A). We found that the relative amount of BrdU incorporation was similar in each of the S-phase gates (Supplemental Fig. 1). The DNA was isolated from each sorted fraction of cells and BrdU-labeled nascent replication intermediates were enriched by immunoprecipitation (Tanaka et al. 2011) with a BrdU antibody (Hansen et al. 2010). The nascent BrdU-labeled sequences from each S-phase fraction were then subjected to next-generation sequencing on the Illumina HiSeq 2000 platform. For each S-phase fraction, ∼5 million reads were uniquely mapped back to release five of the *Drosophila* genome assembly.

The distribution of sequence reads across the genome from each S-phase fraction was indicative of when those genomic sequences were replicated during S phase (Fig. 1B). Importantly, the enrichment of sequence reads in the early (E) and late S-phase (L) fractions were largely nonoverlapping, indicating distinct early and late replicating domains in the *Drosophila* genome (Supplemental Fig. 2). In contrast, the early-mid (E–M) and late-mid (L–M) fractions were less distinct and largely paralleled the early or late fractions, respectively. Together, these results suggest that the *Drosophila* genome is partitioned into domains that replicate during either early or late S phase.

In order to compare the replication timing program across the different cell lines and with our prior array-based replication timing profiles from HU-synchronized cells, we generated continuous replication timing profiles for each cell line (Fig. 1C; Supplemental Fig. 3). To calculate the replication timing profile, we used the ratio of sequence reads at each position (10-kb windows) along the chromosome for each of the four timing fractions. Comparison of the replication timing values, at the whole genome level, between cell lines revealed a strong correlation (Fig. 1D) and these profiles were also correlated with our earlier array-based work (Supplemental Table 1). Recent descriptions of mammalian replication timing profiles (Hansen et al. 2010; Hiratani et al. 2010; Ryba et al. 2010) have also noted a strong correlation between replication profiles derived from different cell lines and lineages. Together, these results suggest that the metazoan replication timing program may be relatively invariant or hardwired into the chromatin landscape.

### Chromatin landscape as a function of the replication program

**Figure 2.**
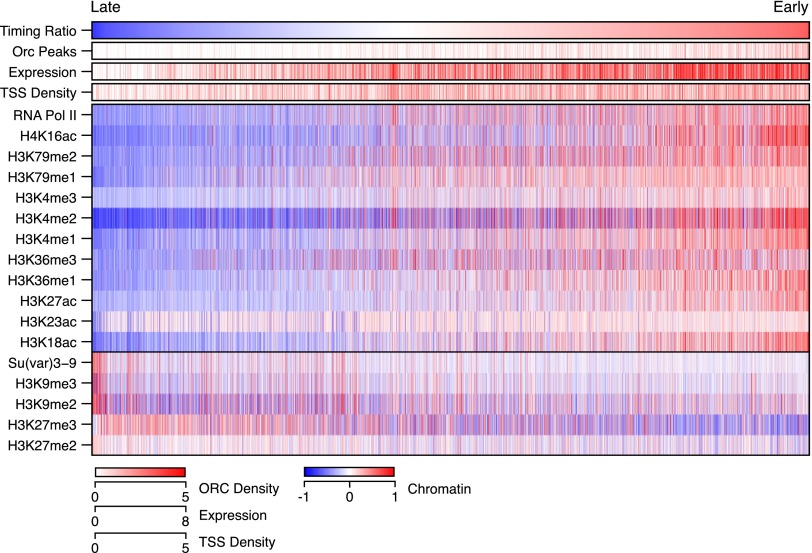
Replication timing correlates with the chromatin landscape. The relative replication timing values from S2 cells were binned into 10-kb windows and ordered from late (*left*, blue) to early (*right*, red). Also plotted are normalized enrichments for gene expression, DNA-binding proteins, and histone modifications as well as ORC density and promoter density. Histone marks and DNA-binding proteins were ordered by row according to their genome wide co-association (determined by k-means clustering of genome-wide correlations, k = 2). Equivalent data for the other two cell lines are available in Supplemental Figure 4.

The wealth of modENCODE data provided an unprecedented opportunity to identify chromatin marks and DNA-binding proteins (The modENCODE Consortium et al. 2010; Kharchenko et al. 2011) associated with early and late replicating sequences in the *Drosophila* genome. For each cell line, we divided the genome into nearly 12,000 10-kb bins and ordered the bins by their replication time from latest (blue) to earliest (red) replicating. We then generated a matrix depicting the relative replication timing, ORC density, promoter density, gene expression, enrichment of 15 chromatin modifications, and two DNA-binding proteins associated with either active transcription (RNA Pol II) or constitutive heterochromatin [Su(var)3-9] for each of the ordered 10-kb replication timing bins (Fig. 2; Supplemental Fig. 4). We found that early replicating sequences were associated with “activating” chromatin marks, ORC binding, high gene density, and gene expression. In contrast, late replicating sequences were typical in gene-poor regions of the chromosomes and marked by an absence of “activating” chromatin marks. Repressive chromatin exists as either facultative or constitutive heterochromatin defined by H3K27me3 and H3K9me2/3, respectively. We found that H3K27me3 and H3K9me2/3 enrichment were both associated with late replicating sequences. H3K27me3 broadly tracked with late replicating sequences, whereas H3K9me3 was associated with a smaller subset of sequences likely associated with the pericentric heterochromatin (Riddle et al. 2011).

**Figure 3.**
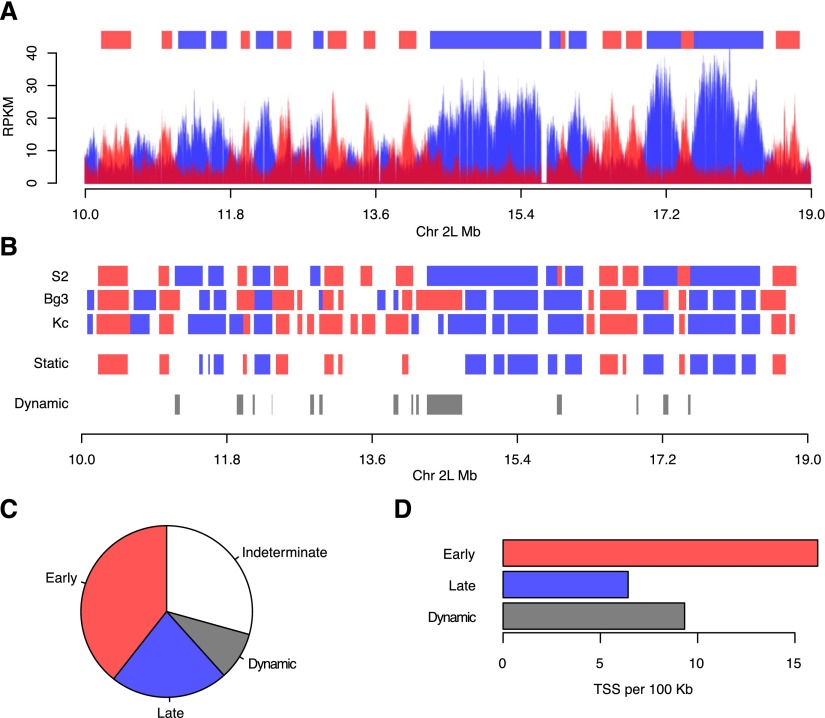
Identification of static and dynamic replication domains. (*A*) Segmentation of the *Drosophila* genome into discrete early and late replicating domains. A three-state hidden Markov model representing early, late, or indeterminate replication timing was used to define early (red) and late (blue) replicating domains (*top*) from the RPKM data from the early and late S-phase fractions. A 9-Mb portion of chromosome 2L is depicted for S2 cells. (*B*) Identification of static and dynamic replication domains. The early and late replication domains were compared between cell lines. Domains that maintain their replication timing in all cell lines (static) or those that change their replication timing in at least one cell line (dynamic) were identified. (*C*) Pie chart representing the fraction of static early (red, 39.3%), static late (blue, 22.2%), dynamic (gray, 8.1%), and indeterminate (30.4%, white) replication timing domains. (*D*) Density of TSS as a function of domain length (per 100 kb) for static early (red), static late (blue), and dynamic (gray) replication domains.

To simplify the comparisons between the different cell lines, we segmented the genome into discrete early and late replicating domains. Because there was little overlap between those sequences that replicated early in S phase and those that replicated in late S phase, we sought to utilize a probabilistic framework to define early and late replicating domains. Specifically, we used a three-state hidden Markov model (Rabiner 1989; Durbin et al. 1998) to partition the *Drosophila* genome into nonoverlapping early and late replicating domains (Fig. 3A; Supplemental Fig. 5). We were able to assign distinct early and late replicating domains to >60% of the genome. Comparison of the distribution of early and late replicating domains in each of the cell lines identified domains that were invariantly early or late in all of the cell lines (Fig. 3B, static) as well as dynamic replication domains that transitioned from early to late or vice versa between cell lines (Fig. 3B, dynamic). In accordance with the similarity in replication timing profiles across the cell lines, the majority of domains were static early (39.3%) or static late (22.2%) and <8.1% of the genome exhibited dynamic differences in replication timing between the cell lines (Fig. 3C). We found that nearly two-thirds (63.77%) of the annotated *Drosophila* genes resided in the static early replicating domains and as a consequence static early replicating domains had the highest density of transcription start sites (TSS). In contrast, the static late domains had a low density of TSS. Interestingly, the dynamic domains, those that change their replication timing between cell lines, also exhibited a low density of TSS similar to the static late domains (Fig. 3D). Finally, we noted that the dynamic domains were enriched for pathways with metabolic gene ontologies including retinol metabolism (*P* < 2.34 × 10^−7^; all *P*-values Bonferroni corrected), metabolism of xenobiotics (*P* < 1.45 × 10^−4^), androgen and estrogen metabolism (*P* < 1.56 × 10^−4^), drug metabolism (*P* < 1.99 × 10^−4^), ascorbate and aldarate metabolism (*P* < 2.61 × 10^−4^), porphyrin and chlorophyll metabolism (*P* < 2.89 × 10^−4^), and starch and sucrose metabolism (*P* < 5.82 × 10^−4^) (Supplemental Table 2). The enrichment of metabolic gene ontologies in the dynamic domains suggests that the replication program may respond to altered transcription during metabolic stress.

**Figure 4.**
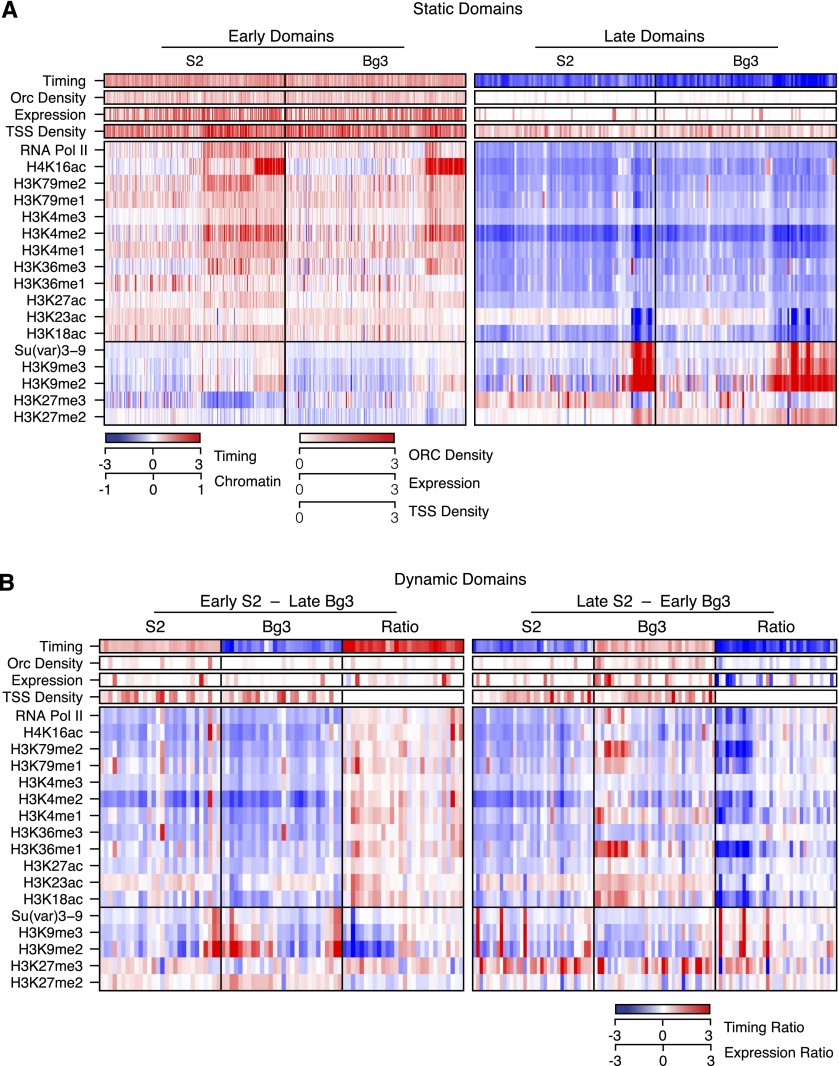
The chromatin environment defines early and late replication domains. (*A*) Static replication domains. The chromatin landscapes for static early (*left*) and late (*right*) domains for S2 and DmBg3 (Bg3) cells are shown. For each domain (columns), the median score for 15 histone modifications and two DNA-binding proteins were determined across the domain region, grouped into three clusters (via k-means analysis), and plotted along with the replication timing ratio (log_2_ difference in RPKM between early and late fractions), normalized expression, and ORC and TSS density (counts per 10 kb). The row order is the same as in Figure 2. (*B*) Dynamic replication domains. Same as in *A*, but grouped into those that switch from early in S2 to late in DmBg3 (*left*) or those that switch from late in S2 to early in DmBg3 (*right*). The difference ratio (S2/DmBg3) for each feature is also represented. Each panel is grouped (via k-means clustering) according to the difference in chromatin marks for differentially early or late replicating regions.

To better understand the defining chromatin features of early and late replicating domains, we examined gene expression, gene density, ORC density, RNA Pol II, and Su(var)3-9 occupancy, along with 15 chromatin modifications for each discrete early or late replicating invariant domain (Fig. 4A). Here we have focused on the distribution of chromatin marks for early and late replicating domains derived from S2 and DmBg3 cells as there are a larger number of chromatin marks available. However, similar trends were also observed for Kc167 cells, which have a less extensive set of profiled chromatin marks (Supplemental Fig. 6). As expected from our ranking based on the calculated replication timing values (Fig. 2), active chromatin marks were enriched in the early replicating domains and depleted in late domains. In concordance with the distribution of chromatin marks, we found that gene expression was highest in the static early domains. In contrast, we found late replicating static domains associated with low gene expression and an enrichment of repressive chromatin marks including H3K9me2/3 and H3K27me3. Finally, we observed a marked difference in the density of ORC-binding sites between the static early and late replicating domains. Although we observed fewer ORC-binding sites in static late domains, the average ORC ChIP-signal at individual origins was similar between the early and late replicating domains (Supplemental Fig. 7), suggesting that the difference in ORC density is not a consequence of antibody accessibility. Together, these results suggest that early and late replicating domains are defined, in part, by the potential to establish an origin of replication (ORC density) which is dependent on chromatin accessibility.

A small subset of replication domains was dynamic and exhibited cell line-specific replication properties (for example, an early domain in S2 that transitioned to a late replicating domain in DmBg3 or vice versa). These domains with the potential to switch their replication timing provided an opportunity to identify key chromatin determinants that modulate the plasticity of the replication timing program. We found that dynamic replication domains had a low gene density and a stereotypical repressive chromatin signature including a low density of ORC binding sites. Analysis of the S2 chromatin marks for replication domains that were early in S2 and late in DmBg3 exhibited few activating chromatin marks and had moderate levels of facultative heterochromatin (H3K27me3).We observed a similar distribution of marks for these domains in DmBg3 cells, even though these regions were late replicating. Based on the dramatic differences in chromatin marks between the static early and late replicating domains (Fig. 4B), we were surprised that there was not a more prominent difference in chromatin marks between the cell lines in the dynamic domains. It should be noted that when we examined the relative ratio of chromatin marks between S2 and DmBg3 cells for those domains that were early replicating in S2 and late replicating in DmBg3, there was a slight increase for activating marks over repressive marks. Similar trends were observed for late replicating S2 domains that transitioned to early in DmBg3 cells. Together, these results indicate that there exists a minimum threshold of activating chromatin marks that are sufficient to transition a late replicating domain to early replicating and that the transition from early to late is dependent on the activation of a limited number of potential origins marked by ORC.

### Male-specific early replication of the X chromosome

**Figure 5.**
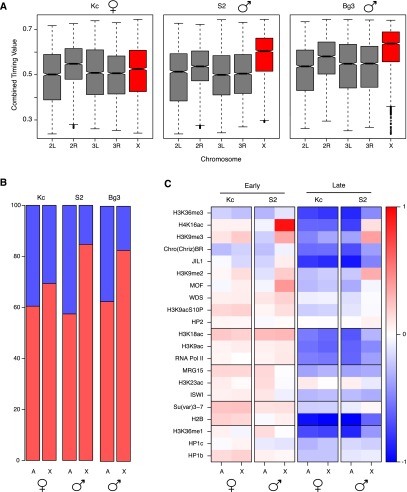
Differential replication timing of the male X chromosome. (*A*) Boxplots representing the distribution of timing values for each chromosome calculated in 10-kb bins. In the male cell lines, the X chromosome replicates significantly earlier than the autosomes (*P*-value < 2.2 × 10^−16^). (*B*) Distribution of early (red) and late (blue) replicating domains for both the autosomes and X chromosome in the three cell lines. (*C*) Heatmap representing the median enrichment score of chromatin marks and DNA-binding proteins found within early (*left*) and late (*right*) replicating domains on the X chromosome or the autosomes (A) in male (S2) or female (Kc167) cells. Red represents an enrichment of log_2_(1) or greater and blue represents a depletion of log_2_(1) or greater.

To identify potential chromosome-specific patterns of DNA replication, we also examined the distribution of replication timing ratios for each chromosome in the different cell lines (Fig. 5A). We found few differences in the distribution of replication timing ratios across each of the chromosomes with the exception of 2R and the X chromosome. Chromosome 2R replicated slightly earlier in all three cell lines, likely due to the higher gene density on this chromosome, a characteristic that has been previously linked to earlier replication timing (Belyakin et al. 2010). The X chromosome replicated significantly earlier than the autosomes (Fig. 5A, red), but this increase in early replication was limited to the male cell lines (S2 and DmBg3). Analysis of the extent of chromosome coverage by the discrete early and late replicating domains also indicated that the early replication domains were expanded on the X chromosome in the male cell lines (Fig. 5B; Supplemental Fig. 8). The sex-specific early replication of the X had first been noted >50 yr ago using tritium-labeled metaphase chromosome spreads (Berendes 1966; Meer 1976) and more recently was rediscovered by our group and others (Schwaiger et al. 2009) in the genomic era.

*Drosophila* males possess one X chromosome whereas females have two. In order to compensate for the difference in gene dosage between the sex chromosome and the autosomes, transcription of the X chromosome is up-regulated approximately twofold in males (Meller and Kuroda 2002; Gupta et al. 2006). This regulation is mediated by the DCC, which is a ribonucleoprotein complex consisting of five proteins (MSL1, MSL2, MSL3, MOF, and MLE) and two noncoding RNAs (*roX1* and *roX2*). The DCC, which is specifically targeted to the X chromosome, hyperacetylates H4K16 via the histone acetyltransferase (HAT) activity of MOF (Conrad and Akhtar 2012). The increase in H4K16 acetylation allows for the up-regulation of X-specific gene expression via RNA Pol II recruitment (Conrad et al. 2012). The male-specific early replication of the X chromosome suggests that the replication and transcription programs are likely responding to the same chromosomal cues—namely an X chromosome-specific increase in H4K16 acetylation levels.

We examined the median enrichment of different chromatin marks in the early and late replicating domains in Kc167 and S2 cells. We found that there was an X chromosome-specific hyperacetylation of H4K16 in both the early and late replicating domains in the male S2 cells (Fig. 5C). These results are consistent with a prior report establishing a strong correlation between hyperacetylation of H4K16 and an advancement of replication timing for the X chromosome (Schwaiger et al. 2009). However, a direct causal link between the DCC-mediated hyperacetylation of H4K16 and the advancement of replication timing on the X chromosome was lacking in the prior study.

### The DCC is necessary for preferential early replication of the X chromosome

**Figure 6.**
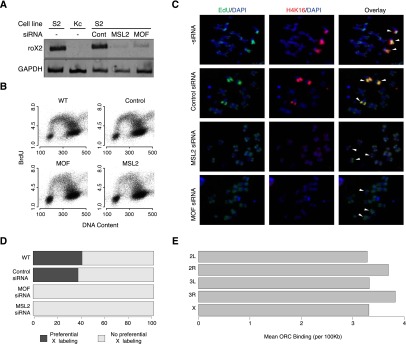
DCC-mediated H4K16 hyperacetylation promotes origin activation on the male X chromosome. (*A*) Depletion of MSL2 or MOF destabilizes the DCC. MSL2 or MOF was depleted in male (S2) cells using dsRNA, and the stability of the DCC was assessed by RT-PCR of the *roX2* noncoding RNA. *GAPDH* served as a control. (*B*) Depletion of the DCC knockdown does not impair the cell cycle. The cell cycle profiles of MOF or MSL2 siRNA-treated cells are similar to those of untreated cells or cells treated with control siRNA. (*C*) A functional DCC is required for the preferential replication of the X chromosome. Wild-type and dsRNA-treated cells were pulse-labeled with EdU (green) after release from HU arrest and then allowed to complete S phase before being arrested in metaphase. White arrowheads designate the X chromosomes. (*D*) Quantitation of the data in panel *C*. At least 100 metaphase spreads were counted for each condition. (*E*) Hyperacetylation of the X chromosome does not increase the selection of potential origins (ORC binding). The density of ORC binding (per 100 kb) in S2 cells is plotted for each chromosome.

To establish a causal and direct relationship between DCC-mediated hyperacetylation of H4K16 on the X chromosome and advancement of replication timing, we sought to deplete key DCC components by RNAi and assess the impact on the replication program. Male S2 cells were treated with dsRNA targeted toward MSL2 and MOF, two key components of the DCC, or a control dsRNA derived from the pUC19 plasmid (Fig. 6A). Reduced expression of either DCC component causes destabilization of the complex (Gorman et al. 1993), resulting in the degradation of the two noncoding RNAs *roX1* and *roX2*. Previously, Schwaiger and colleagues attempted to deplete MOF via RNAi in S2 cells (Schwaiger et al. 2009); however, upon reduction of MOF, the cells stopped proliferating and there was a marked decrease in the number of S-phase cells. Importantly, depletion of either DCC component (MOF or MSL2) did not affect the cell cycle in our studies, and S phase remained unperturbed following RNAi treatment (Fig. 6B).

To assess the impact of the DCC and H4K16ac on the DNA replication program, we first sought to cytologically monitor the production of early replication intermediates in control cells and those depleted by RNAi for either MOF or MSL2. DCC-depleted and control cells were first synchronized at the G1/S boundary by HU treatment. The cells were then released from the G1/S arrest and briefly pulsed with EdU for 10 min to label actively replicating sequences. The cells were allowed to progress through S phase before being arrested in the subsequent metaphase by the microtubule destabilizing agent colcemid. Immunofluorescence was used to cytologically assess the location and chromosomal distribution of EdU-labeled replication intermediates and H4K16ac along the mitotic figures (Fig. 6C). Depletion of either DCC component, MOF or MSL2, resulted in a loss of X chromosome-specific H4K16 hyperacetylation as expected. It should be noted that the H4K16 hyperacetylation occurs on multiple different mitotic figures, and this is due to the inherent aneuploidy of the S2 genome, which features a large number of chromosomal copy number variations as well as structural rearrangements and translocations (Zhang et al. 2010; Lee et al. 2014). Control cells and those treated with a nonspecific scrambled siRNA (pUC19) exhibited preferential early EdU incorporation on the X chromosome (40.6% and 37.3% of metaphase spreads, respectively), while no such enrichment was detected in DCC knockdown cells (Fig. 6D). These results demonstrate that the DCC-mediated H4K16 hyperacetylation is not only correlated with early replication, but is required for the male-specific early replication of the X chromosome in *Drosophila*.

The DCC and hyperacetylation of H4K16 are necessary for the male-specific early replication observed on the X chromosome. The DCC-mediated X chromosome-specific hyperacetylation of H4K16 may advance replication timing by promoting origin selection (ORC binding), origin activation, or facilitating replication fork movement. In order to determine whether the earlier replication of the male X chromosome is due to an increase in origin selection, we have examined the distribution of ORC-binding sites on the autosomes and the X chromosome in male S2 cells (Fig. 6E). We found no significant difference in the density of ORC-binding sites between the X chromosomes and the autosomes. These data suggest that the earlier replication of the X chromosome is regulated at the level of origin activation (or fork movement) rather than at the level of origin selection.

## Discussion

Metazoan chromosomes are organized into distinct domains of similar replication timing. Sequences within these broad chromosomal domains, spanning tens to hundreds of kilobases, are largely replicated at similar times during S phase. The defining features of these replication timing domains are poorly characterized at the molecular level, but are correlated with transcription, chromatin modifications, and chromosome organization (Pope et al. 2013). Here we describe the replication timing program of three *Drosophila* cell lines in the context of extensive modENCODE chromatin data. We found that the *Drosophila* replication timing program is “hardwired” and relatively similar across cell lines. Strikingly, the few regions of the genome that are dynamic and change replication timing between cell lines are gene poor and tend to be susceptible to minor increases in activating marks. ORC density in these dynamic domains does not change between cell lines, suggesting that changes in replication timing between lineages are regulated at the level of origin activation rather than origin selection. Finally, we find that a sex-specific difference of H4K16 acetylation is necessary for the early replication of the X chromosome.

We found that the majority (60%) of the *Drosophila* genome was partitioned into stable early and late replicating domains, which were conserved among the three cell lines. Early replicating domains were clearly demarcated by the presence of activating chromatin marks, increased gene density, transcriptional activity, and elevated ORC binding. In contrast, late replicating domains were defined by an absence of activating marks, low gene density, decreased ORC binding, and the presence of constitutive or repressive facultative heterochromatin, H3K9me2/3 and H3K27me2/3 respectively. Together, these results suggest that origin selection (ORC binding) within early or late replication timing domains is a critical factor in establishing the replication timing program.

ORC binding to the DNA appears to be mediated by chromosome accessibility. Prior work in *Saccharomyces cerevisiae*, *Drosophila*, and mammalian cells established that nucleosome occupancy was a key determinant of ORC localization (Berbenetz et al. 2010; Eaton et al. 2010; Lubelsky et al. 2011). The decreased ORC density in late replicating regions is likely due to decreased DNA accessibility in these regions of repressive chromatin. Consistent with this hypothesis, analysis of DNase hypersensitivity data throughout the *Drosophila* genome also revealed decreased accessibility in late replicating regions (Kharchenko et al. 2011). Similarly, experiments in mammalian systems also identified a strong link between DNA accessibility and replication timing with late replicating regions being resistant to nuclease digestion (Takebayashi et al. 2012). Although comprehensive ORC localization data does not exist for mammalian cells, it is reasonable to speculate that mammalian late replicating domains will also have a decreased density of ORC association. Additional factors such as the interaction of ORC1 with H4K20me2 (Kuo et al. 2012) and ORCA with H4K20me3 (Shen et al. 2010), a chromatin mark often associated with repressive chromatin, may assist in the recruitment of ORC into highly inaccessible heterochromatic regions.

The decreased density of ORC will result in fewer potential replication origins in the late replicating domains. Activation of replication origins is regulated in part by CDK and DDK activities during S phase (Heller et al. 2011). These CDK substrates and DDK activity are limiting for origin activation during S phase and their overexpression can promote the early activation of normally late replicating origins in *S. cerevisiae* (Mantiero et al. 2011; Tanaka et al. 2011). We expect that similar mechanisms will regulate the temporal activation of metazoan origins of replication. The increased density of ORC in discrete early replicating domains relative to late replicating domains may function as a sink for limiting CDK targets or DDK activity to these regions. Thus, the likelihood of an origin activation event during early S phase will be significantly higher in those regions of the genome marked by elevated ORC levels. As S phase progresses and more of the early replication domains are replicated, the likelihood of activating late origins will be increased. This may account for the near dichotic distribution of early and late replicating domains in the *Drosophila* genome.

As precursor cells differentiate during metazoan development their chromatin organization changes to accommodate the lineage-specific patterns of gene expression. Mammalian studies of the DNA replication program have identified cell-type-specific changes in the replication timing program (Hansen et al. 2010; Ryba et al. 2011). Our identification of dynamic replication domains and the depth of the *Drosophila* modENCODE data afforded us the opportunity to identify chromatin signatures associated with the change within a domain from early to late replication timing or vice versa. Surprisingly, we did not observe dramatic changes in the chromatin environment at regions that switched replication timing. Instead we found a very slight change in activating and repressive chromatin marks that were associated with the switch from late to early or early to late, respectively. The most striking feature of dynamic replication domains was that they possessed a chromatin environment that would normally be associated with late replication. Specifically, these dynamic domains exhibited an absence of activating chromatin marks and low gene density and ORC binding. These results suggest that the default time of replication for low gene density intergenic regions occurs late in S phase and that subtle changes in chromatin structure and or DNA accessibility permit them to become early replicating. We did not observe a change in ORC binding between cell lines in those dynamic replication timing domains, suggesting that origin activation instead of origin selection is a driving determinant of re-programming the DNA replication program.

Dosage compensation mediated by sex chromosome-specific epigenetic environments presents a unique opportunity to understand how the chromatin environment impacts the DNA replication program. In mammals, dosage compensation is achieved by the inactivation of one of the female X chromosomes. The inactive copy of the X chromosome is condensed and silenced (Schulz and Heard 2013) and replicates later in S phase than does the active X chromosome (Gartler et al. 1992). In contrast, transcription along the single male X chromosome in *Drosophila* is up-regulated approximately twofold to match the expression levels of the autosomes (Meller and Kuroda 2002; Gupta et al. 2006). The increase in X-specific transcriptional activity is controlled by the DCC, a nucleoprotein complex which hyperacetylates H4K16 (Gelbart and Kuroda 2009). Not only is the male X chromosome differentially transcribed, but early cytological studies also revealed that it is differentially replicated (Berendes 1966; Lakhotia and Mukherjee 1970). More recently, genome-wide approaches have also demonstrated that the male X chromosome is differentially replicated. Not surprisingly, the male-specific early replication of the X chromosome is correlated with H4K16ac (Schwaiger et al. 2009). Here, we were able to extend this correlation to a causal relation by demonstrating that hyperacetylation of H4K16 by MOF is required for the early replication of the X chromosome. Importantly, the specificity of H4K16 hyperacetylation on the X chromosome and the X-specific advancement of replication timing suggest a direct effect on the replication program rather than a secondary effect of altered transcription.

In *Drosophila* females, the actions of the DCC are blocked by the expression of sex lethal (SXL), which inhibits the translation of MSL2 (Bashaw and Baker 1995; Kelley et al. 1997). Without MSL2, the DCC is unstable. The loss of SXL results in the expression of MSL2, which leads to the stabilization and targeting of the DCC to the X chromosome (Gorman et al. 1993), resulting in the hyperacetylation of the X chromosome. We tested if H4K16ac was sufficient for early replication of the X chromosome by depleting SXL in female Kc167 cells. Although we did occasionally observe X-specific hyperacetylation of H4K16 on the X chromosome in female cells following depletion of SXL, we were unable to conclusively determine if elevated H4K16Ac was sufficient for the early X-specific replication (data not shown).

Although we expected that depletion of SXL and stabilization of the DCC in females would cause an increase in transcription of the X chromosome and early replication, several studies have shown that H4K16ac may not be the only regulating factor of X chromosomal activity. For example, depletion of SXL with RNAi in female Kc167 cells only results in a modest increase of transcriptional activity, not the near twofold increase over autosomal genes observed in the male S2 cell line (Alekseyenko et al. 2012). Additionally, an X-linked reporter did not show increased expression upon stabilization of the DCC (Sun et al. 2013). These results suggest that hyperacetylation of the X chromosome by MOF may prime the X chromosome for increased transcription, but additional regulatory factors may be required to achieve the full increase in gene expression. Therefore, the hyperacetylated state of the chromatin is not sufficient for increased transcription and thus may not be sufficient for early replication of the X chromosome.

We did not observe a sex-specific difference in ORC density between the autosomes and the X chromosome. The fact that ORC density did not change strongly suggests that the earlier replication of the male X chromosome driven by H4K16 hyperacetylation is due to a higher rate of replication initiation rather than by an increased density of potential origins (ORC-binding sites). Alternatively, H4K16ac may facilitate replication fork progression downstream from replication initiation. In support of increased initiation of DNA replication, the Schubeler group noted increased origin activity on the male X chromosome (Schwaiger et al. 2009). Our results demonstrate that the increased origin activity is downstream from origin selection and that H4K16ac may increase the accessibility of replication origins to critical initiation factors.

## Methods

### BrdU labeling and FACS sorting

Cells were plated at a concentration of 2 × 10^6^ cells/mL in Schneider’s insect medium (Invitrogen) and allowed to grow overnight before adding 10 µg/mL BrdU (Roche 280879) for 1 h. The cells were harvested and resuspended in 600 µL of PBS and fixed by adding 10 mL of cold 70% EtOH dropwise with slow vortexing and incubating at −20°C for at least 30 min. The ethanol was removed, and the cells were resuspended in 20 mL of ice cold PBS and allowed to rehydrate for at least 1 h at 4°C. A small sample was labeled with FITC-conjugated anti-BrdU antibodies (BD Pharmigen 556028) and analyzed using the Becton Dickinson FACScan instrument to verify the incorporation of BrdU. The rest of the cells were labeled with 10 µg/mL of Propidium Iodine and sorted into four S-phase fractions based on their DNA content using the BD DiVa flow cytometer and cell sorter (Becton Dickinson).

### Immunoprecipitation of BrdU-labeled DNA

Sorted cells were resuspended in SDS-PK buffer (50 mM Tris•Cl pH8, 10 mM EDTA, 1 M NaCl, 0.5% SDS), supplemented with 50 µg/mL glycogen and 0.2 mg/mL proteinase K to a final concentration of 5 × 10^6^ cells/mL and lysed by incubating for 2 h at 56°C. Two-hundred microliters (equivalent to 10^6^ cells) from each fraction was used for the IP. Two-hundred microliters of SDS-PK buffer, supplemented with 50 µg/mL glycogen, was added to the lysed cell extract, and the DNA was extracted once with phenol chloroform and once with chloroform and precipitated in isopropyl alcohol. The precipitated DNA was resuspended in 500 µL of TE, sonicated to a size of ∼800 bp (15 min, 30 sec on, 30 sec off on high setting using the Bioruptor sonicator [Diagenode, UCD-200]), and denatured by incubating at 95°C for 5 min. The denatured DNA was transferred to a fresh tube containing 60 µL of 10× IP buffer (0.1 M Na•phosphate pH7, 1.4 M NaCl, 0.05% Triton X-100). Forty microliters of 12.5 µg/mL (1:40 dilution in PBS of BD Biosciences #555627) of anti-BrdU antibody was added and the reaction was incubated for 20 min on a rotator before adding 7.2 µL of anti-rabbit IgG (Sigma #M-7023) and rotating for an additional 20 min. The DNA-antibody pellet was washed once in 1× IP buffer and resuspended in 200 µL of digestion buffer (50 mM Tris•Cl pH8, 10 mM EDTA, 0.5% SDS, 250 ng/mL proteinase K) and digested overnight in a 37°C incubator. An additional 100 µL of digestion buffer was added, and the reaction was incubated for an additional hour at 56°C. The precipitated DNA was extracted once with phenol/chloroform and once with chloroform and precipitated by adding 1 µL of 20 mg/mL glycogen, 100 µL of 10 M ammonium acetate, and 750 µL of EtOH. The precipitated DNA was resuspended in 40 µL of H_2_O, of which 30 µL was used to prepare sequencing libraries.

### Illumina sequencing libraries

Libraries were prepared as previously described (Lubelsky et al. 2012), using Illumina TruSeq adaptors. Libraries were multiplexed and sequenced on the Illumina HiSeq 2000. Sequence reads were aligned to release five of the *Drosophila* genome using Bowtie (Langmead et al. 2009). All experiments were performed with two independent biological replicates.

### RNA interference

Double-stranded RNAs (dsRNAs) were generated using PCR products flanked by a T7 promoter sequence at each end as a template for in vitro transcription using the T7 RiboMAX Express RNA Production System (Promega P1320). The complementary RNA strands were annealed to generate dsRNA. S2 cells were grown in Schneider’s medium supplemented with 10% fetal bovine serum (FBS) and 1% penicillin/streptomycin/glutamine (Invitrogen) at 25°C. For RNA interference (RNAi), cells were washed and plated in serum free Schneider’s medium at a concentration of 2 × 10^6^ cells/mL, and 10 µg/mL of dsRNA was added. After incubation for 1 h at 25°C, 2× medium was added to a final concentration of 1 × 10^6^ cells/mL. Cells were incubated for 6 d. To ensure that the cells continued to cycle and did not arrest at a high cell density, the cell concentration was maintained at 1 × 10^6^ cells/mL, and the dsRNA concentration was maintained at 5 µg/mL.

### RT-PCR

RNA was extracted from cells using the RNeasy Kit (Qiagen 74104) according to the manufacturer’s instructions and treated with RQ1 DNase (Promega M6101). Reverse transcription was done using the iScript cDNA Synthesis Kit (Bio-Rad 1708891). Briefly, 500 ng of RNA was used for cDNA synthesis. From each reaction, 2 µL of cDNA was used as a template for PCR using the following primers: *roX2*: AGCTCGGATGGCCATCGA, CGTTACTCTTGCTTGATTTTGC; *GAPDH* (Igaki et al. 2002): CCACTGCCGAGGAGGTCAACTA, GCTCAGGGTGATTGCGTATGCA.

### EdU labeling and immunofluorescence

An intra-S-phase checkpoint arrest was induced by adding 1 mM hydroxyurea (HU) (Sigma H8627) for 24 h. The cells were then washed twice and replated in fresh medium. The cells were pulsed with 10 µM EdU (Invitrogen A10044) for 10 min, washed twice, and replated. Cells were then allowed to incubate at 25°C for 6 h. To arrest the cells in metaphase, 3 µg/mL of demecolcine (Sigma D1925) was added to the cells for 8 h before harvesting. To create metaphase spreads, cells were harvested and resuspended in 0.8% sodium citrate at a concentration of 5 × 10^5^ cells/mL for 8 min. The cells were then placed in a single chamber cytofunnel (Shandon) and deposited onto slides using the CytoSpin4 (Thermo Scientific). The slide chambers were centrifuged at 2000 rpm with high acceleration for 10 min at room temperature. The cells were then treated with 4% paraformaldehyde in PBS for 5 min and washed with PBS. To detect H4K16ac, we used a rabbit anti-H4K16ac antibody (Millipore 07-329) at a concentration of 1:100 and a secondary goat anti-rabbit 568 fluorescent antibody (Invitrogen A-11011) at a concentration of 1:500. To detect EdU, we used the Click-iT EdU Alexa Fluor 488 Imaging Kit (Invitrogen C10337) according to the manufacturer’s instructions. DNA was detected with Vectashield containing DAPI (Vector Laboratories H-1200).

### Computational methods

#### Data accession numbers

All genomic data are publicly available at the NCBI Gene Expression Omnibus (GEO; http://www.ncbi.nlm.nih.gov/geo/). The following accessions were utilized: GSE17281, GSE17279, GSE17280, GSE20888, GSE20889, GSE20887, GSE41349, GSE41350, GSE41351.

#### Short read alignment

Sequenced reads were aligned to the dm3 release 5.12 of the *D. melanogaster* genome assembly (downloaded from the UCSC Genome Browser, and available at ftp://hgdownload.cse.ucsc.edu/goldenPath/dm3/bigZips/chromFa.tar.gz), using the software package Bowtie (version 0.12.7) (Langmead et al. 2009); along with the –best, –try-hard, and –strata parameters, the following options were used: The number of acceptable within-seed mismatches was limited to two per read, and seed length was set to 20 nt. Reads with more than one valid alignment were ignored. All replicates were combined prior to alignment. See Supplemental Table 3 for alignment statistics.

#### Generating continuous replication timing profiles

For each fraction, RPKM (reads per thousand kb, per million mappable reads) was calculated over nonoverlapping bins of 10 kb, and a weighted average between the four fractions was determined. Weights were selected such that reads from the latest fraction would have a score between 0 and 0.25, late-mid fractions 0.25–0.5, early-mid fractions 0.5–0.75, and reads from the earliest fraction would have a score between 0.75 and 1.0; the weights assigned to each fraction represents the midpoints of each of these ranges: 0.125, 0.375, 0.625, and 0.875 for early, early-mid, late-mid, and late fractions respectively. Each bin was then assigned a score using the formula (where *x* represents a vector of RPKM for the four fractions, and *w* represents the vector of weights):
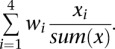


Finally, to account for regions containing spurious amplifications or low coverage, the resulting signal was interpolated over ∼200 kb via local polynomial regression (implemented through the R programming environment using the “loess” function).

#### Generation of discrete early and late replication timing domains

To identify discrete early and late replicating domains, we trained a three-state hidden Markov model (HMM, representing early, late, or indeterminate replication timing), with each state emitting a bivariate normal distribution, corresponding to the joint distribution of RPKM from the early and late fractions.

#### Signal normalization

Within each cell line, and for the early and late fractions, RPKM was determined over bins of 5 kb, stepping every 1 kb. Because RPKM normalization standardizes differences in library size, it can obscure the contribution of each individual fraction. To ensure the proportional representation of each chromosome, we weighted the resulting scores by the mean RPKM across all fractions per chromosome; additionally, to avoid any effects of spuriously amplified sequences, we set the maximum possible bin score to the 95th percentile.

#### Initial model generation and training

To empirically determine the emission parameters for each state of the HMM, k-means (k = 3) analysis was performed over the log_2_ ratio between early and late replication timing signals, establishing genomic loci from which the means and covariance between the early and late signals were derived. Transition probabilities were set to favor very high self-transition probabilities, and the initial probabilities were set to be equal between the three states. Subsequently, for each chromosome, training was performed via the iteration of the Baum-Welch algorithm, until the improvement in the log-likelihood between subsequent iterations was <0.05 (Supplemental Table 4). See Supplemental Tables 5 and 6 for the resulting state emissions and transitions. Following training, the Viterbi algorithm was used to classify genomic regions (bins) into one of the three states. In order to compensate for the kinetics of BrdU incorporation and the rate of replication, we set a lower bound on the smallest number of contiguous states to be 40 bins (∼45 kb); all contiguous bins less than this were subsequently merged, then assigned to the largest continuous neighboring state. All computational analyses were carried out via the R programming environment (Hornik 2013), using the RHmm package to implement the HMM modeling, training, and assessment (Taramasco and Bauer 2012).
